# ­­­ **­­­**Recent advances in managing Peyronie’s disease

**DOI:** 10.12688/f1000research.9041.1

**Published:** 2016-09-26

**Authors:** Oliver Kayes, Rauf Khadr

**Affiliations:** 1 St James Hospital, Leeds, LS9 7TF, UK

**Keywords:** peyronie's disease, penile curvature, PD treatment

## Abstract

Peyronie’s disease remains an under-reported and debilitating problem which can result in significant physical and psychological symptoms for some men. The classic symptom complex includes penile curvature, penile plaque, and penile pain. Men can also present with erectile dysfunction, penile instability, and penile shortening, alongside feelings of low mood/libido, dysmorphobia, and low self-esteem. This review highlights the current key publications in the medical literature and provides updates on new clinical therapies whilst postulating about potential future treatments on the horizon.

## Background

Peyronie’s disease (PD) is a common source of both outpatient referral and treatment dilemma for urologists. Prevalence is reported to be in the region of 3% ^1^. Traditionally, men present with a new-onset curvature of the erect penis, which may be associated with pain in the initial (active) phase. PD is frequently associated with erectile dysfunction and penile shortening. Finally, there may be a palpable plaque of disease, typically on the dorsal aspect of the penis. The natural history of the disease suggests stabilisation (quiescent phase of 12 to 18 months) with resolution of pain and a permanent penile deformity. Improvement in the curvature may occur spontaneously in a minority of patients ^2^. It affects men principally between the ages of 40 and 60 and is associated with diabetes mellitus, Dupuytren’s contracture, and plantar fascial contracture (Lederhosen’s disease). There may be a preceding history of trauma ^3^.

The aetiology of the disease is thought to be repeated minor microvascular trauma during intercourse, resulting in intra-tunical bleeding and subsequent inflammation and fibrosis. Interestingly, we have seen PD in sexually naïve men who have never had penetrative intercourse. This may not refute the hypothesis but points further to a multifactorial process. Transforming growth factor beta is thought to exacerbate the irregular healing response. Histologically, there is excessive connective tissue, increased cellularity, and random orientation of collagen fibres within the Peyronie’s plaque. Subsequently, the dysfunctional tunical tissue restricts normal expansion of the underlying corpus cavernosum, creating the observed curvature. There may be flaccidity distal to the lesion with or without wasting/hourglass deformity or rotation in more severe cases ^3^.

Surgical correction is traditionally the mainstay of treatment, although there is associated morbidity, most commonly in terms of infection and haematoma in the early post-operative period, and later penile shortening, erectile dysfunction, recurrent curvature, and glans hypoesthesia. Therefore, remedial intervention is undertaken with a degree of reluctance in most men seeking corrective surgery. This can result in delayed definitive treatment, often causing anxiety and dissatisfaction in patients because of impairment of their sexual health. As a result, there has been extensive research into non-surgical interventions that may be implemented earlier in the disease process, thus improving patient quality of life and reducing surgical morbidity.

The aim of this review article is to summarise recent advances in PD treatment, including surgical, non-surgical, and pharmacological interventions. A literature review was performed by using a Medline review of publications from 2013 onwards, focusing on randomised controlled trials where available.

## Intralesional therapy

### Collagenase

Despite the existence of early studies dating back to 1982 ^4^, collagenase clostridium histolyticum was approved by the US Food and Drug Administration in only 2013 as Xiaflex™ (Xiapex™ in the European market) for patients with a deformity of at least 30 degrees and palpable disease. Treatment comes in the form of an injection of two collagenases which act synergistically to cleave tropocollagen. The regimen involves up to four cycles of treatment 6 weeks apart, each cycle requiring two injections of 0.58 mg separated by 24 to 72 hours. Patients are also educated in penile modelling to occur three times daily. Approval was gained following the published data from IMPRESS (Investigation for Maximal Peyronie’s Reduction Efficacy and Safety Studies) I and II trials, identical phase III placebo controlled studies ^5^. These studies of 551 men evaluated reduction in curvature and Peyronie’s Disease Questionnaire (PDQ) score as their main outcome measures, and 401 patients made up the final modified intention-to-treat analysis. Curvature was reduced by 17 and 9 degrees (34% versus 18%) in the treatment and placebo arms, respectively. PDQ bother and symptom scores were reduced by -2.8 versus -1.8 and -2.9 versus -1.3, respectively, favouring the treatment group. Serious adverse events were noted in six patients, of whom four (three corporeal rupture and one haematoma) required surgery. All corporeal ruptures were related to intercourse. Overall, there does appear to be at least a modest benefit and it remains the most promising non-surgical therapy. The cost per cycle is £1300; hence, the treatment is currently not available routinely within the National Health Service but is being evaluated by the National Institute for Health and Clinical Excellence. Modification of the trial protocol to reduce the total number of injections and cycles alongside the use of a traction device appears to represent improved health economics and we await availability of published data to that effect.

### Verapamil

The role of calcium channel blocker verapamil in PD is thought to be due to an upgrade of collagenase activity and inhibition of extracellular collagen transport ^6^. Whilst both intra-lesional and electromotive drug administration (EMDA) verapamil have been shown to provide some benefit in historic studies, direct comparison has not been previously reported. A randomised trial by Mehrsai *et al*. compared route of treatment in 60 patients undergoing an injection once per week for 6 weeks ^7^. The authors found no significant difference in reduction of plaque size alongside a noted improvement in erectile function (albeit not significant). Penile pain assessed by visual analogue scale was significantly reduced in the EMDA group compared with the injection group (-4.1 versus -1.8) at 3 months. Penile curvature was improved in both groups, and increased shift towards a less-than-30-degree group was noted especially in the EMDA category. This improvement, however, was not quantified in more detail or statistically significant. Adverse events were not reported. They concluded that EMDA was at least a comparable and less invasive treatment modality. Notably, the study excluded patients with curvature of greater than 45 degrees and those unable to achieve penetrative intercourse. Recent guidelines from Europe and the US do not recommend routine use of EMDA verapamil ^8, 9^.

Intra-lesional verapamil has also been assessed alongside tadalafil by Dell’Atti *et al*. in a randomised trial ^10^. The study compared verapamil (12-weekly) and tadalafil daily alone and in combination. No significant improvement in curvature was noted in any group over the course of a 3-month study period. There was improvement in pain for all groups, most notably in the combination arm; however, statistical significance was not achieved. Only improvement in International Index of Erectile Function (IIEF) score showed significance in the combination group (14.4 versus 18.2 versus 23.1). The authors acknowledge that small numbers limit their study; however, the role of daily tadalafil may augment more traditional strategies ^10^.

### Interferon α-2B

First reported by Duncan *et al*. in 1991, interferon therapy was shown to affect collagen in PD by inhibiting fibroblast proliferation and promoting collagenase activity *in vitro* ^11^. There is a paucity of randomised trial data during this period of review, and the last significant trials were conducted in 2006.

These data concluded that interferon provided only modest benefit in conjunction with significant side effects ^12, 13^. A retrospective review by Trost *et al*. ^14^ indicated a 54% response rate in 127 patients but a mean improvement in curvature of only 9 degrees. A further review in 2015 from Stewart *et al*. reported a 91% response rate but with similar modest improvement in curvature ^15^. The treatment is currently included in both the American Urological Association and European Association of Urology guidance.

## Pharmacotherapy

### Vitamin E and antioxidants

Vitamin E has been the subject of numerous historical trials and the general consensus is that its antioxidant properties improve pain during the acute phase, but not curvature. The most recent controlled trial, by Paulis *et al*., studied the role of vitamin E 600 mg daily in augmenting a standard treatment protocol of combined intra-lesional/transdermal verapamil, non-steroidal anti-inflammatory drugs, and herbal antioxidants ^16^. The group receiving vitamin E demonstrated global reduction in curvature in 97% versus 48% of patients in the control group. The noted reduction was modest but statistically significant: mean of 12.2 degrees versus 6.7 degrees. Significant improvements were noted in the treatment group in terms of plaque size as well as subgroup analysis of IIEF scores. Medium-term follow-up of these patients demonstrated durability of these findings and further supported combination therapy ^17^.

A further randomised trial, by Favilla *et al*., showed significant improvement in IIEF scores for patients given antioxidants alongside intra-lesional verapamil, compared with verapamil alone ^18^. No improvement in curvature was noted.

Overall, there is limited evidence to support the use of vitamin E and antioxidants other than as an adjunct to more recognised therapies.

### Tamoxifen and Potaba

There are multiple oral agents promoted for use in the early phase of PD development. Typically, tamoxifen and Potaba fall victim to problems similar to those of alternatives such as colchicine and acetyl- l-carnitine because of a lack of recent randomised data to support their routine use. Both treatments are endorsed by international guidelines for improvement in penile pain but not specifically for reduction in curvature. Park *et al*. report on Potaba versus combination therapy of tamoxifen, l-carnitine, and tadalafil ^19^. Perhaps the most striking result was that two-thirds of the patients in the Potaba arm withdrew for varying reasons, although treatment side effects were cited as the largest single factor. Resolution of pain was once again significant, and more so in the combination group. Sixty-six percent of patients overall noted an improvement in curvature of 30% or more; however, this does include the large number of dropouts.

## Non-surgical intervention

### Extracorporeal shockwave therapy

Extracorporeal shockwave therapy (ESWT) remains second- or third-line treatment in PD, and decreased efficacy has been highlighted in a single-blinded randomised trial by Hatzichristodoulou *et al*. ^20^ Similarly, a significant reduction in pain was reported (85% versus 48% in the placebo group). However, no improvement was witnessed in plaque size and curvatures worsened by up to 40% of patients in the treatment group. The authors conclude that the role of ESWT can no longer be justified in the treatment of PD.

### Vacuum tumescence and traction devices

No randomised data exist for these treatment modalities. Objective improvement has been seen in a recent controlled study of traction devices, as have significant improvements in curvature, erectile function, and pain. Importantly, 40% of patients avoided surgical intervention ^21^. These may be a useful adjunct to treatment, although larger studies are needed and the daily traction time required (up to 8 hours) may prove to be a limiting factor. However, as highlighted in the first section, these devices may have a renewed role in penile modelling techniques with plaque dissolution seen in collagenase-style therapies.

## Surgical intervention

There have been no significant advances in PD surgical management in terms of randomised controlled data. In keeping with historical trends, there have been numerous retrospective reviews of modifications to traditional plication and grafting techniques. Suffice to say, plication remains the standard for patients without erectile dysfunction and a curvature of less than 60 degrees provided that the associated loss of length is not problematic. Incision and grafting are indicated in patients falling outside these criteria, although plaque excision without grafting is reported as a simplified technique ^22^.

Inflatable penile prosthesis and manual remodelling have been re-assessed in a retrospective review by Chung *et al*. comparing choice of manufacturer ^23^. They found high satisfaction (79%) and 5-year mechanical survival (87% or greater) in both AMS CX and Coloplast Titan devices as well as revision and complication rates of below 10%. Further results regarding implant insertion and simultaneous plication demonstrated similar levels of satisfaction ^24^. Implant surgery remains the mainstay of treatment for patients with significant curvature and erectile dysfunction.

More data are emerging regarding surgery following failed intra-lesional collagenase treatment, suggesting that there is no additional operative complexity following previous local therapy ^25^.

## Psychological aspects and patient-reported outcomes in Peyronie’s disease

Traditionally, patients with PD are counselled well regarding the natural course of the disease and morbidity associated with the relevant treatment options. Less attention is given to what may be a significant psychological impact of the disease and its diagnosis. A review by Nelson and Mulhall in 2013 ^26^ identified emotional difficulties and clinical depression in up to 81% and 48% of patients, respectively. Relationship difficulties were reported in 54%. Levine also draws attention to this, in particular the degree of curvature not being “definitively linked to the degree of psychological distress” and the need for a multifaceted team-based approach ^27^.

Hellstrom *et al*. developed and implemented the PDQ questionnaire during the IMPRESS trials ^28^. They assessed the validity of this tool in 2013 and found its three domains addressing symptoms, pain, and bother useful and reliable in categorising disease impact. Coyne *et al*. further analysed the questionnaire in terms of responsiveness and also found it to be an effective tool ^29^. Whilst functional assessments such as the IIEF questionnaire are relatively commonplace in urological clinics, Peyronie’s-specific questionnaires may have a separate role in highlighting patients at risk of psychological harm.

## The future

Recent publication of a single-arm interventional study investigating the role of intra-lesional hyaluronic acid in PD represents a low-cost and low-side-effect avenue for intervention ^30^. Improvements in curvature, IIEF score, and sexual function were noted, supporting the need for further study.

As with many other fields of medicine, stem cell treatment remains an exciting prospect in regeneration of healthy tissue ^31^. Within this subject, the research remains largely laboratory based, although in the era of individualised treatment plans this may change.

## Conclusions

Intra-lesional treatments with modelling show that the most promise is treating PD non-surgically and at an earlier stage. Generally, high treatment costs in non-insurance-based health systems, heterogenous experience, and the lack of widespread availability impede the current evidence base, particularly in terms of randomised trials.

Currently, we believe that patients receive appropriate counselling regarding the nature of the disease and treatment morbidity. Undoubtedly, we are failing to generate a greater emphasis with regard to the psychosexual, mood-altering, and relationship aspects of PD. Improved tools to assess patient depression, systemic health issues, and markers of poor quality of life remain to be fully implemented in routine practice. PD remains a difficult condition to successfully treat and should remain the remit of dedicated sexual health specialists and surgeons if we are to improve outcomes for men and women affected by PD.
